# Effects of Fruit Shading on Gene and Protein Expression During Starch and Oil Accumulation in Developing *Styrax tonkinensis* Kernels

**DOI:** 10.3389/fpls.2022.905633

**Published:** 2022-06-02

**Authors:** Qikui Wu, Hong Chen, Zihan Zhang, Chen Chen, Fangyuan Yu, Robert D. Guy

**Affiliations:** ^1^Collaborative Innovation Centre of Sustainable Forestry in Southern China, College of Forest Science, Nanjing Forestry University, Nanjing, China; ^2^State Forestry and Grassland Administration Key Laboratory of Silviculture in Downstream Areas of the Yellow River, College of Forestry, Shandong Agricultural University, Tai’an, China; ^3^Department of Forest and Conservation Sciences, Faculty of Forestry, University of British Columbia, Vancouver, BC, Canada; ^4^State Key Laboratory of Tree Genetics and Breeding and Key Laboratory of Tree Breeding and Cultivation, State Forestry Administration, Research Institute of Forestry, Chinese Academy of Forestry, Beijing, China

**Keywords:** fruit shading, kernel development, carbon partitioning, oil biosynthesis, differentially expressed proteins

## Abstract

*Styrax tonkinensis* has great potential as a biofuel feedstock source having industrial oilseeds with excellent fatty acids (FAs) composition and good fuel properties. Photosynthesis in the developing pericarp could affect the carbon distribution in kernel. During kernel development, more carbon sources are allocated to starch rather than lipid, when the pericarp photosynthesis is reduced by fruit shading treatment. After shading the fruits at 50 days after flowering (DAF), samples of shaded fruit (FSK) and controls (CK) were collected at 80 DAF and analyzed using the proteomic method. We identified 3,181 proteins, of which 277 were differentially expressed proteins, all downregulated in the FSK group. There were 56 proteins found involved in carbohydrate metabolism and lipid biosynthesis leading to oil accumulation with their iTRAQ ratios of FSK/CK ranging from 0.7123 to 1.1075. According to the qRT-PCR analyses, the key genes related to FA and triacylglycerol (TAG) biosynthesis were significantly downregulated between 60 and 90 DAF especially at 80 DAF, while the key genes involved in starch biosynthesis and FA desaturase had no significant difference between the two groups at 80 DAF. Fruit shading is a negative treatment for lipid accumulation but not starch accumulation by restraining enzymic protein expression involved in FA and TAG biosynthesis during *S. tonkinensis* kernel development.

## Introduction

Autotrophic plants acquire carbon through photosynthesis, which provides organic compounds for conversion into different metabolites (Ashraf and Harris, 2013). Photosynthate produced in chloroplast-containing “source” tissues is transported to different “sink” tissues or organs to be consumed in respiration or utilized in the synthesis of starch, oil, and proteins (Bennett et al., 2011). The transport of photosynthates from source to sink is one of the most important factors affecting seed development (Li et al., 2011; Hua et al., 2012). Different light intensity caused by shading treatment resulted in significant differences in metabolites in blueberry leaves (Wu et al., 2022). Photosynthesis in the pericarp contributes a certain proportion of the source carbon deposited in kernels and affects seed quality and kernel nutrient accumulation (Xu et al., 2016). During fruit development, treatments used to manipulate the photosynthetic rate can affect the carbon distribution in kernels, especially between starch and lipid (Ma et al., 2014; Zhang et al., 2018).

*Styrax tonkinensis* (Pierre) Craib ex Hartwich, as a woody biodiesel species, has seed kernels with high oil content (more than 50%) during seed maturation (Wu et al., 2019a). Oleic acid (C18:1) and linoleic acid (C18:2) are the two major fatty acids (FAs) and account for more than 80% of the total FA content (Zhang et al., 2017). During kernel development, the biological metabolic processes of starch and FA biosynthesis are catalyzed by two series of enzymes; glucose-1-phosphate adenylyltransferase (AGP), starch synthase (glgA) and 1,4-alpha-glucan branching enzyme (glgB) play important roles in starch synthesis (Lin et al., 2006; Bourgis et al., 2011), while the pyruvate dehydrogenase complex (PDC), acetyl-CoA carboxylase complex (ACC), 3-oxoacyl-ACP reductase (KAR), enoyl-ACP reductase (EAR), acyl-CoA:DAG acyltransferase (DGAT), and phospholipid:diacylglyceryl transferase (PDAT) take on major responsibilities in FA and triacylglycerol (TAG) biosynthesis (Bates et al., 2013; Niu et al., 2015).

Mechanisms of oil accumulation have been analyzed at the molecular level using transcriptomic and proteomic methods in many oilseed plants, including *Elaeis guineensis* (Bourgis et al., 2011; Silva et al., 2014), *Vernicia fordii* (Zhang et al., 2014; Zhan et al., 2016), *Jatropha curcas* (Grover et al., 2014; Liu et al., 2015) and *S. tonkinensis* (Wu et al., 2020, 2021). In our previous studies, ACC and PDC were placed in the center of the regulatory network for oil accumulation during *S. tonkinensis* kernel development (Wu et al., 2020, 2021). However, at the protein level, the biological functioning of TAG-related enzymes, such as DGAT and PDAT, is unclear, as these endoplasmic reticulum-associated proteins appear at middle stages during kernel development (Wiese et al., 2007; Liu et al., 2018). According to the nutrient distribution in the continuum of the pericarp, seed coat, and kernel, the nutrient deposition center would be transferred into kernels from the maternal units (the pericarp and seed coat) which may function as a nutrient buffer storage area between the mother tree and the kernel (Bennett et al., 2011; Wu et al., 2019b).

In another previous study, we found that pericarp photosynthesis in *S. tonkinensis* was stimulated by treatment with the brassinosteroid hormone 24-epibrassinolide and attenuated by fruit shading (Zhang et al., 2018). Oil accumulation in developing *S. tonkinensis* kernels was markedly impaired at about 80 days after flowering (DAF) while the fruits grew in low light conditions under three layers of black, non-woven fabric bags (Shade#3 treatment). Meanwhile, a much larger proportion of carbon precursor was directed toward starch accumulation causing a high starch/oil ratio in the developing kernels under this treatment. A possible reason may be that the oil-related enzymes, such as ACC, DGAT, and PDAT, are more susceptible to carbon source shortage.

To further understand the effect of fruit shading on developing kernels at protein levels, we repeated the Shade#3 treatment on developing *S. tonkinensis* fruits to analyze changes in differentially expressed proteins (DEPs) involved in kernel development and nutrient distribution by the isobaric tags for relative and absolute quantification (iTRAQ) method. The aims of the present study were to: (i) assess proteomic profiles of samples of shaded fruit (FSK) and controls (CK) kernels; (ii) identify DEPs between the two groups; (iii) identify the proteins involved in starch and lipid biosynthesis; and (iv) illucidate the molecular mechanism of carbon partitioning under source shortage during *S. tonkinensis* kernel development.

## Materials and Methods

### Plant Material

The study was performed on 20 eight-year-old *S. tonkinensis* trees (from Jishui, Jiangxi Province) at the Styracaceae Germplasm Repository (32°32′ N, 118°50′ E) situated in Nanjing, China, belonging to the north subtropical monsoon humid climate zone. The plants grew under natural conditions with moderate soil fertility. In accordance with our previous study (Zhang et al., 2018), we chose 20 infructescences from each tree for fruit shading using three layers of black, non-woven fabric bags (Shade#3) on 18 July 2018 (50 DAF). No leaves were included in the shading bags. Twenty untreated fruits from the same trees were labeled as a control group. For each group, fresh fruits were collected every 10 days from July 18 (50 DAF) to August 27 (90 DAF). At each sampling, shaded and unshaded fruits were randomly selected from each tree and pooled into four biological replicates (i.e., fruits from five trees per replicate). To adjust for their growth, the number of fruits collected per treatment per tree was reduced from five to three over the sampling period. Fruits were placed on dry ice for transport to the laboratory and, after removing the pericarps and seed coats, kernels were transferred to liquid nitrogen storage for iTRAQ and qRT-PCR analyses.

### Protein Extraction and iTRAQ Analysis

Kernels collected from FSK and CK groups at 80 DAF were used for proteomic analysis, again with four biological replicates per treatment. Protein extraction and digestion were performed according to our previous study (Wu et al., 2021). The peptide mixture resulting from trypsin digestion was labeled using an 8-plex iTRAQ kit (AB Sciex, Framingham, United States) following the manufacturer’s instructions and Ye et al. (2010). Peptides were labeled as follows: FSK samples with iTRAQ-113, 114, 115, and 116, CK samples with iTRAQ-117, 118, 119, and 121. The labeled peptides were pooled before lyophilization in a vacuum concentrator.

Reverse-phase high-performance liquid chromatography (HPLC) was used to separate labeled peptides following procedures as described by Liu et al. (2017). The peptide mixture was re-dissolved in solvent A (20 mM ammonium formate adjusted to pH 10.0 with NH_4_OH), and then fractionated by high pH separation using an Ultimate 3,000 liquid chromatography system (Thermo Fisher Scientific, Waltham, United States) connected to an XBridge C18 reverse-phase column (4.6 mm × 250 mm, 5 μm) equilibrated with solvent B (20:80 (v/v) ammonium formate buffer (pH 10.0):acetonitrile). After separation, 12 final fractions were collected and vacuum-dried for the next step. Re-dissolved peptides were analyzed by Nano-HPLC-MS/MS using an EASY nLC 1,000 system (Waters Corporation, Milford, United States) coupled to a Q-Exactive mass spectrometer (Thermo Fisher Scientific, Waltham, United States). The parameter settings were as described by our previous study (Wu et al., 2021).

After analyzing the tandem mass spectra with PEAKS Studio version 8.5 (Bioinformatics Solutions, Waterloo, Canada), customized protein sequence databases derived from transcriptome sequence analysis (Wu et al., 2020) were searched with PEAKS DB assuming trypsin as the digestion enzyme and with fragment ion mass and parent ion tolerances set at 0.05 Da and 7 ppm, respectively. The bioinformatics analyses were specified according to Lu et al. (2019). After protein identification, the DEPs between FSK and CK groups were recognized if their abundance ratios were over 1.5 or <0.667 with *p* < 0.01 (by ANOVA) and they contained at least two unique peptides. Identified proteins were classified using the public protein databases. Proteins involved in carbohydrate metabolism and lipid biosynthesis were subsequently tallied *via* the gene functional annotation.

### Total RNA Extraction and qRT-PCR Analysis

Quantitative real-time PCR (qRT-PCR) of seven genes related to carbon flux from sucrose to fatty acids at five time points from 50 to 90 DAF was carried out. Total RNA extraction, first-strand cDNA synthesis, and amplification primer design (Supplementary Table 1) were performed as described in our previous study (Wu et al., 2020). All reactions were done on a StepOne Real-Time PCR System using SYBR Green Dye (Applied Biosystems, Foster City, United States; Takara, Dalian, China). Relative gene expression was evaluated using the 2^−ΔΔCt^ method with 18S ribosomal RNA as an internal control.

## Results

### Mass Spectrometry Analysis and Protein Identification

**Figure 1:**
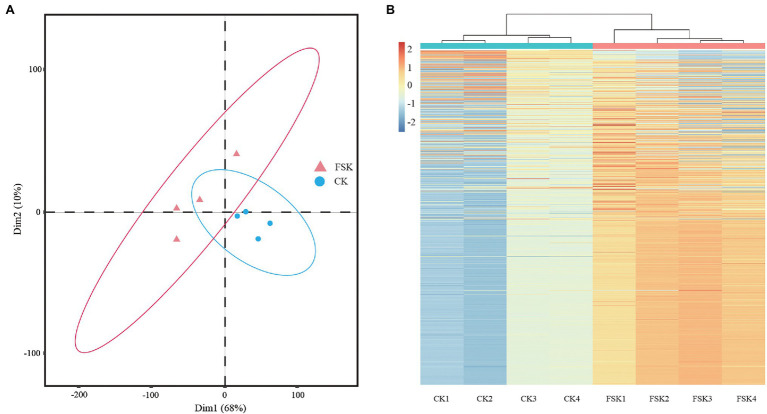
Quality control of samples of different groups. **(A)** Principal component analysis of samples. **(B)** Hierarchical cluster analysis of samples.

Peptides and proteins of FSK and CK samples at 80 DAF were identified by the iTRAQ method. After searching the unique mass spectra and peptides against the *S. tonkinensis* transcription database, a total of 3,181 proteins were obtained (Supplementary Table 2). The proteomic data from all samples followed a normal distribution and showed high repeatability across biological replicates by principal component (PCA) and hierarchical cluster (HCA) analyses. For the PCA analysis (Figure 1A), all four replicates of the CK group were clustered together near the origin in the lower right quadrant, but well separated from the samples of the FSK group distributed across the other three quadrants. For the HCA analysis (Figure 1B), replicates were clustered together depending on whether the fruit was shaded or not.

The 3,181 identified proteins were annotated against the Gene Ontology (GO) database and then categorized into three functional categories: biological process (BP), cellular component (CC), and molecular function (MF). The top 20 sub-categories of annotated proteins in each term were listed (Supplementary Figure 1). Under the BP term, the major sub-categories were “metabolic process” (1,491), “cellular process” (1,439), and “organic substance metabolic process” (1,314). Nested within the CC term, “cell part” (1,368) and “intracellular part” (1,317) were the major sub-categories. “Catalytic activity” (1,490) and “binding” (1,348) were the major MF groups.

### Identification of Differentially Expressed Proteins

According to the relative expression levels, a total of 227 DEPs between the FSK and CK groups at 80 DAF were identified (Supplementary Table 3). All of the DEPs were downregulated in the FSK group relative to the CK group. To analysis their functions, all DEPs were annotated against GO, Clusters of Orthologous Groups (COG), and the Kyoto Encyclopedia of Genes and Genomes (KEGG) databases.

**Figure 2:**
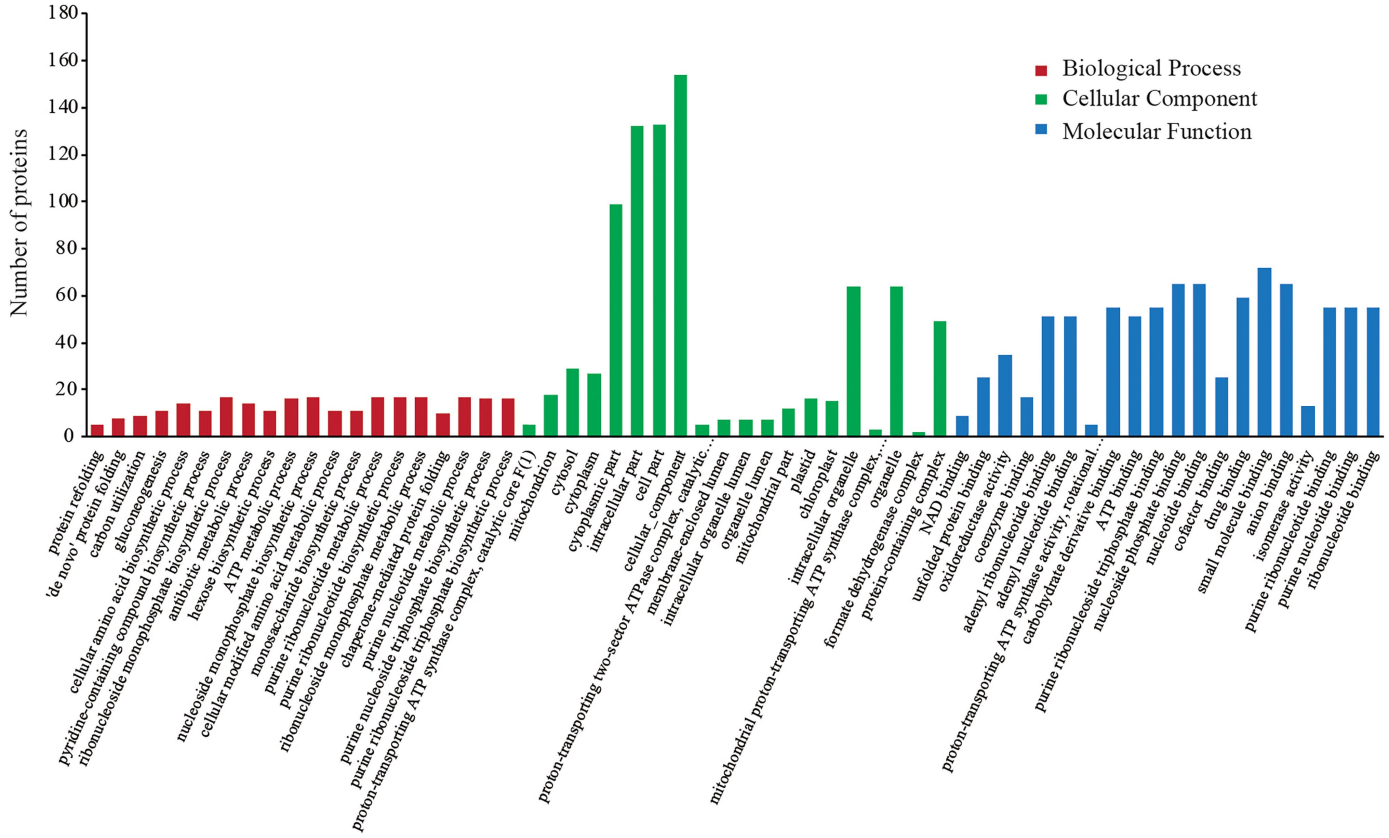
GO annotation analysis of differentially expressed proteins (DEPs).

In the GO annotation analysis, the 227 DEPs were categorized into the same three functional categories (i.e., BP, CC, and MF). The sub-categories of annotated proteins arranged by value of *p* in each term are listed in Figure 2. The DEPs in the BP term were mainly distributed in “protein refolding,” “‘*de novo*’ protein folding,” “carbon utilization,” and “gluconeogenesis.” Under the CC term, the major sub-categories were “proton-transporting ATP synthase complex, catalytic core *F* (1),” “mitochondrion,” “cytosol,” and “cytoplasm.” “NAD binding,” “unfolded protein binding,” “oxidoreductase activity,” and “coenzyme binding” were the major MF sub-categories.

**Figure 3:**
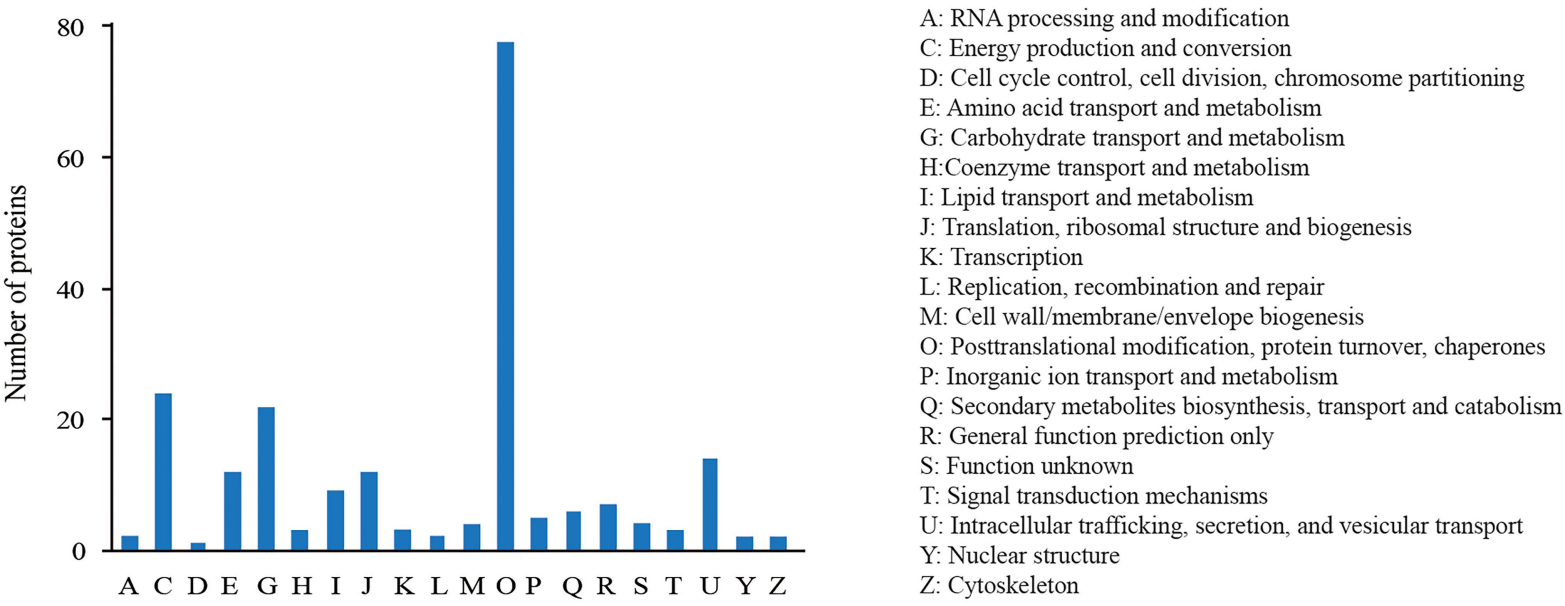
COG annotation analysis of DEPs.

The COG annotation analysis classified the 227 DEPs into 20 categories (Figure 3) with the majority in “post-translational modification, protein turnover, chaperones” (79), followed by “energy production and conversion” (24), “carbohydrate transport and metabolism” (21), and “intracellular trafficking, secretion, and vesicular transport” (15). There were 10 proteins annotated into “lipid transport and metabolism.”

**Figure 4:**
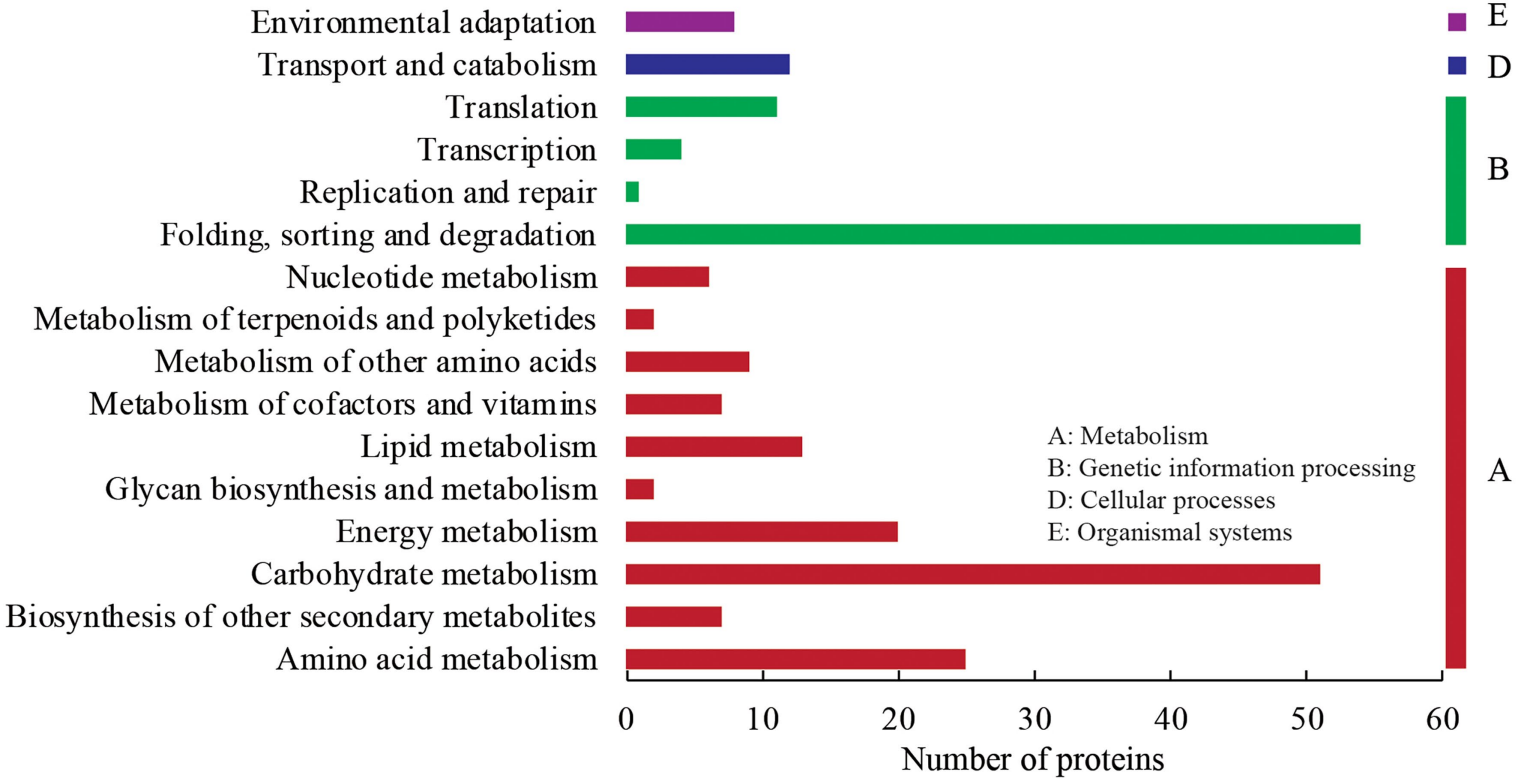
KEGG annotation analysis of DEPs.

In the KEGG annotation analysis, the 227 DEPs were assigned into four KEGG categories (metabolism, genetic information processing, cellular processes, and organismal systems), 16 sub-categories, and 79 KEGG pathways (Figure 4). Under the metabolism term, 51 DEPs were annotated into carbohydrate metabolism with the largest number within “glycolysis/gluconeogenesis” (11), followed by “starch and sucrose metabolism” (7), “amino sugar and nucleotide sugar metabolism” (6), “pyruvate metabolism” (6), and “glyoxylate and dicarboxylate metabolism” (5). Twenty-five, twenty, and thirteen DEPs were annotated into amino acid metabolism, energy metabolism, and lipid metabolism, respectively. Under the genetic information processing category, 54 DEPs were annotated into folding, sorting, and degradation with the majority in “protein processing in endoplasmic reticulum” (35). Additionally, there were 12 and 8 DEPs annotated into “transport and catabolism” (cellular processes) and “environmental adaptation” (organismal systems), respectively.

### Identification of Protein Species Involved in Oil Accumulation

According to the annotation results, 56 of the 3,181 identified proteins are involved in carbohydrate metabolism and lipid biosynthesis leading to oil accumulation (Supplementary Table 4), including five proteins for starch and sucrose metabolism, 12 for glycolysis/gluconeogenesis, three for the pentose phosphate pathway, six for the TCA cycle, 13 for FA biosynthesis, seven for TAG biosynthesis, and three for FA metabolism. The iTRAQ ratios of all 56 proteins between the FSK and CK groups ranged from 0.7123 to 1.1075, with the majority being downregulated. According to the protein annotation, the identified transcription factors (TFs) were mainly concentrated in the PKL, C3H, MYB, and SBP families (Supplementary Figure 2). Most of the identified TFs showed downregulation trends in the Shade#3 treatment.

The ratios of sucrose synthase (SS) and sucrose phosphate synthase (SPS) between the FSK and CK groups were 0.9249 and 0.8741, respectively, with slight and non-significant downregulation in FSK samples. The mean value of the ratios of enzymes involved in starch synthesis was 0.9813, including AGP (0.8381), glgA (0.9984), and glgB (1.1075). The mean values of the ratios of enzymes involved in glycolysis/gluconeogenesis, the pentose phosphate pathway, and TCA cycle were 0.8175, 0.8108, and 0.8237, respectively, most of which showed significant downregulation. The mean value of enzymes related to FA biosynthesis in plastid was 0.7964, with a lower ratio of KAR (0.7369) and EAR (0.7538). The mean value of enzymes involved in TAG biosynthesis in endoplasmic reticulum was 0.8157 with a lower ratio of DGAT (0.7534). The ratio of oil body membrane protein (i.e., OLE) was 0.8922 with slight and non-significant downregulation in FSK samples. The mean value of enzymes involved in oil metabolism was 0.8051 between FSK and CK groups.

### qRT-PCR Analysis of Related Key Genes

**Figure 5:**
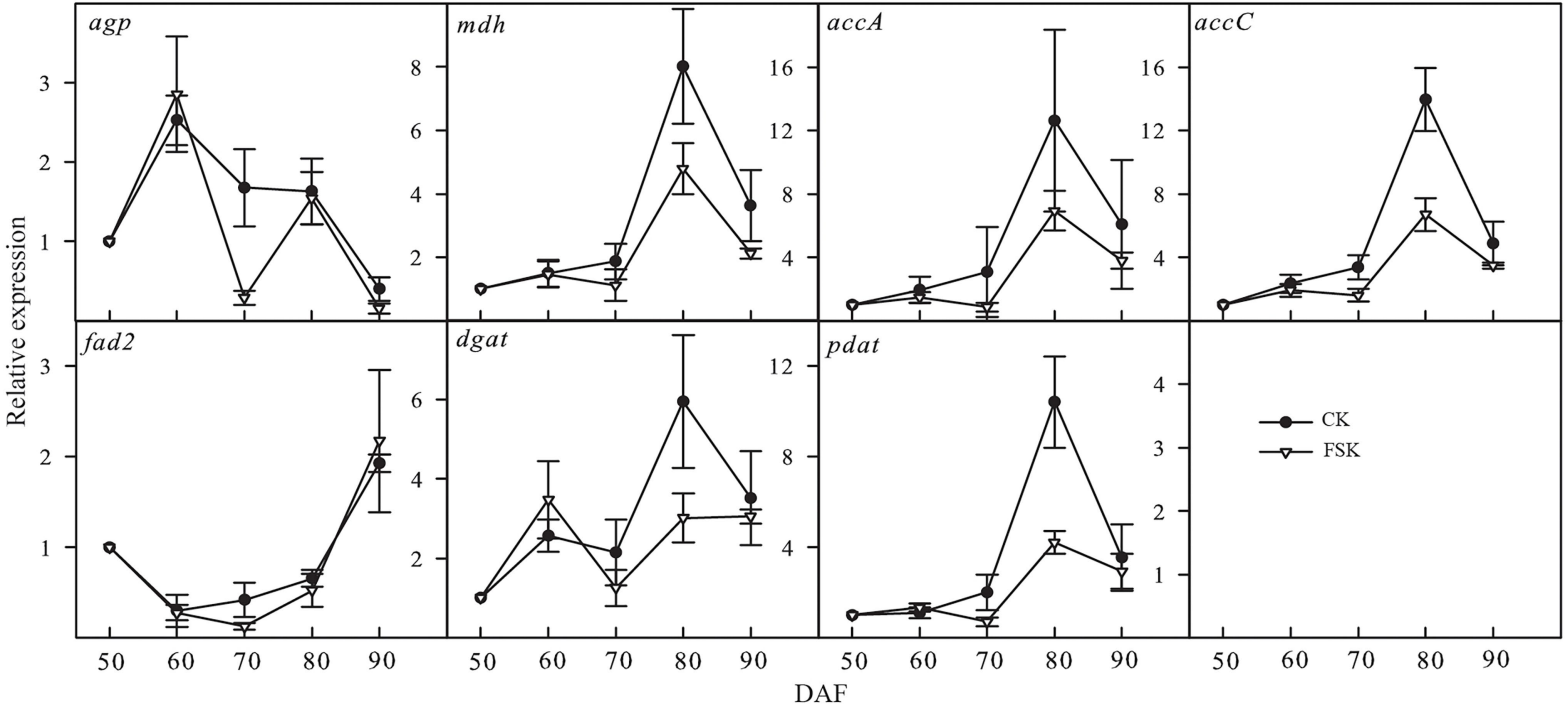
Temporal expression patterns of genes involved in oil accumulation in the FSK and CK groups during *Styrax tonkinensis* kernel development from 50 to 90 DAF. Panels show relative expression levels determined by qRT-PCR of seven key genes (50 DAF as the control).

Based on the results of our proteomic analysis, seven genes (*agp*, *mdh*, *accA*, *accC*, *fad2*, *dgat*, and *pdat*) known to be involved in oil accumulation were chosen for confirmation of mRNA expression levels by qRT-PCR (Figure 5). The dynamic expression of *agp* showed similar trends in the FSK and CK groups except at 70 DAF. The dynamic expression of fatty acid desaturase 2 (*fad2*) was very similar in both the FSK and CK during the whole observation period. The expression of the other key genes varied between 70 and 90 DAF, especially at 80 DAF where there was significantly lower expression in the FSK samples, relative to the CK group.

## Discussion

In higher plants, photosynthesis occurs in all green parts including leaves and other non-leaf organs, such as bracts, pedicels, and pericarps (Aschan and Pfanz, 2003; Kocurek et al., 2015). The change of photosynthesis rate effects the metabolite distribution (Wu et al., 2022). The supply of carbohydrate required by developing seed is affected significantly by treatments, such as fruit shading or pedicel ring cutting, which severely limits the delivery of photosynthate from the maternal plant (Hua et al., 2012). During soybean seed development, pod removal or leaf blade shading increased the protein percentage and decreased the oil content (Proulx and Naeve, 2009). In some plants, the seeds themselves can carry out photosynthesis that positively affects their growth (Andriotis et al., 2012; Galili et al., 2014). The percent oil content in green plant tissues increases with the increase of photosynthesis (Ohlrogge and Jaworski, 1997). In contrast, reduced inputs of source carbon decreases seed biomass accumulation and changes the nutrient composition of kernels by increasing the competitive accumulation of alternative storage compounds (Baud and Lepiniec, 2010).

Zhang et al. (2018) studied the effects of fruit spraying with 24-epibrassinolide and fruit shading treatments on kernel development in *S. tonkinensis*. They found significant changes in the allocation of carbon between starch and fatty acids at about 80 DAF when fruits were shaded by three layers of black, non-woven fabric bags (Shade#3). In our study, *S. tonkinensis* kernels were shaded with Shade#3 treatment from 50 DAF and then collected for iTRAQ-based proteomics analysis at 80 DAF and qRT-PCR analysis at 50, 60, 70, 80, and 90 DAF, respectively. There was high repeatability in the PCA and HCA analyses (Gerhardt et al., 2019). All the replicates from FSK and CK groups clustered together respectively, indicating that *S. tonkinensis* kernel biological processes at the protein level changed significantly with fruit shading. The number of proteins identified in this study (3,181) was more than that (2,338) in the proteome analysis of *S. tonkinensis* kernel samples from different time points (Wu et al., 2021), which may be due to the gradual formation of proteins during kernel development, such as the proteins related to TAG formation in ER. All the 227 DEPs were downregulated in the FSK group, indicating that fruit shading inhibited *S. tonkinensis* kernel development by reducing photosynthesis in the pericarp and decreasing carbon source flow into kernels (Zhang et al., 2018; Wu et al., 2019b). The significant difference of protein expressions was shown at 80 DAF consistent with that the 70–80 DAF stage was identified to be an important time in the nutrient distribution in the continuum of the pericarp, seed coat, and kernel, while the deposition center for different nutrients moves to the kernel from either the pericarp or the seed coat (Wu et al., 2019b).

In developing kernels, the biological processes of oil biosynthesis are complex and involve carbohydrate decomposition, pyruvate, and acetyl-CoA formation, FA carbon chain extension, and TAG formation (Rawsthorne, 2002; Alonso et al., 2011; Lin et al., 2017). Meanwhile, oil accumulation is affected by starch synthesis, energy metabolism, amino acid metabolism (Ekman et al., 2008; Andriotis et al., 2012; Chaitanya et al., 2015). In our study, we identified 56 proteins related to 46 enzymes leading to oil accumulation and 10 of the proteins showed significant downregulation in the FSK group. According to the expression of related proteins in the FSK and CK groups, the expression ratio (FSK/CK) of proteins involved in FA biosynthesis was the lowest (0.7977), while those in starch biosynthesis was the highest (0.9813). In particular, the expression ratios of *glaA* and *glgB*, involved in starch biosynthesis, were 0.9984 and 1.1075 respectively, indicating that the fruit shading had no significant effect on the biological process of starch biosynthesis while simultaneously inhibiting oil accumulation in developing *S. tonkinensis* kernels (Wang et al., 1993; Zhang et al., 2018). For qRT-PCR analysis, *agp* involved in starch biosynthesis showed similar change trends in FSK and CK groups especially at 80 DAF, consistent with the change trend of related enzyme activities in the two groups (Zhang et al., 2018). Key genes involved in FA and TAG biosynthesis, such as *accA*, *accC*, *dgat*, and *pdat,* showed low expression in FSK groups, consistent with the change trends of related enzyme activity and oil content in the two groups. Moreover, the FA composition showed little change under fruit shading, consistent with little change in *fad2* expression at the transcript, protein, and enzyme activity levels (Zhang et al., 2018). The effect of fruit shading on kernel nutritional composition is to increase the relative starch content and decrease oil content by inhibiting the activity of related enzymes involved in oil accumulation.

## Conclusion

In this study, 3,181 proteins and 227 DEPs were identified in samples of shaded fruit and controls kernels of *S. tonkinensis* at 80 DAF using the iTRAQ method. All the DEPs showed downregulated expression patterns in the FSK group. During *S. tonkinensis* kernel development, fruit shading treatment resulted in significant difference in oil content by constraining enzymic protein expression involved in FA and TAG biosynthesis and then leads to the decrease of seed oil content. Furthermore, the relative increase of starch content in shaded fruits is consistent with the observation that fruit shading had no effect on starch-related enzymic protein expression.

## Data Availability Statement

The raw data supporting the conclusions of this article will be made available by the authors, without undue reservation.

## Author Contributions

QW contributed to conceptualization, data curation, investigation, methodology, roles/writing—original draft, and writing—review and editing. HC contributed to data curation, methodology, and software. ZZ contributed to investigation and software. CC contributed to data curation and investigation. FY contributed to conceptualization, funding acquisition, project administration, supervision, and writing—review and editing. RG contributed to writing—original draft and writing—review and editing. All authors contributed to the article and approved the submitted version.

## Funding

This work was supported by the Doctorate Fellowship Foundation of Nanjing Forestry University, Joint Research Project Based on Cooperative Program for Bachelor of Science in Forestry by Nanjing Forestry University and University of British Columbia, National Natural Science Foundation of China (3197140894), and A Project Funded by the Priority Academic Program Development of Jiangsu Higher Education Institutions (PAPD). The funders had no role in the design of the study and collection, analysis, and interpretation of data and in writing the manuscript.

## Conflict of Interest

The authors declare that the research was conducted in the absence of any commercial or financial relationships that could be construed as a potential conflict of interest.

## Publisher’s Note

All claims expressed in this article are solely those of the authors and do not necessarily represent those of their affiliated organizations, or those of the publisher, the editors and the reviewers. Any product that may be evaluated in this article, or claim that may be made by its manufacturer, is not guaranteed or endorsed by the publisher.

## Supplementary Material

Supplementary Figure 1 GO classification of proteins identified during *S. tonkinensis* kernel development.

Supplementary Figure 2 The distribution of identified transcription factors during *S. tonkinensis* kernel development.

Supplementary Table 1 Primer sequences used in the experiments.

Supplementary Table 2 Proteins identified during *S. tonkinensis* kernel development.

Supplementary Table 3 DEPs identified during *S. tonkinensis* kernel development.

Supplementary Table 4 Identification of proteins involved in carbohydrate metabolism and oil accumulation during *S. tonkinensis* kernel development.
