# Supplementary figures and images for: Effects of Fruit Shading on Gene and Protein Expression During Starch and Oil Accumulation in Developing Styrax tonkinensis Kernels
Source: Front Plant Sci. 2022 Jun 2;13:905633. doi: 10.3389/fpls.2022.905633 (PMC9201641; doi:10.3389/fpls.2022.905633)

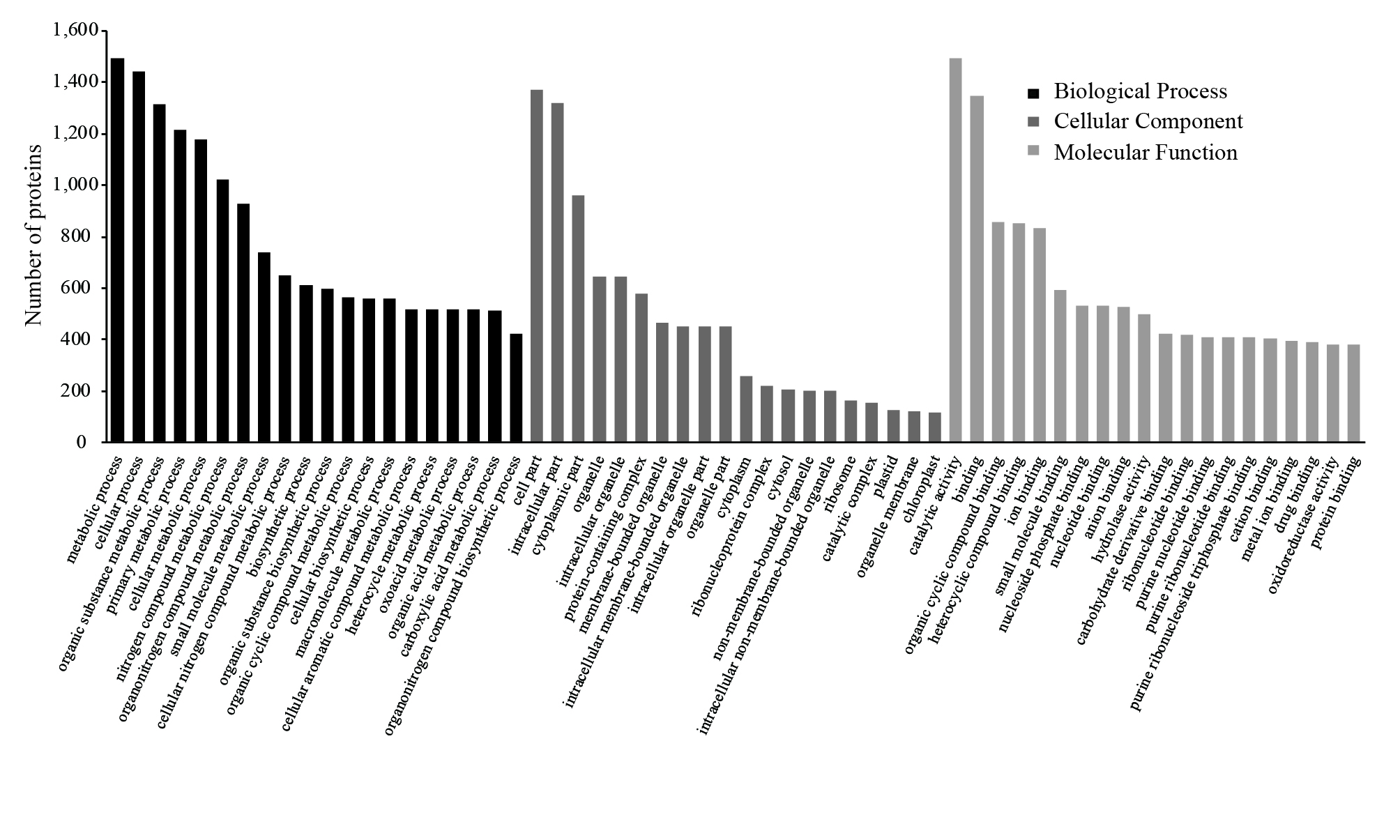

Supplement: Supplementary Figure 1 — GO classification of proteins identified during S. tonkinensis kernel development. [file Image_1.JPEG]

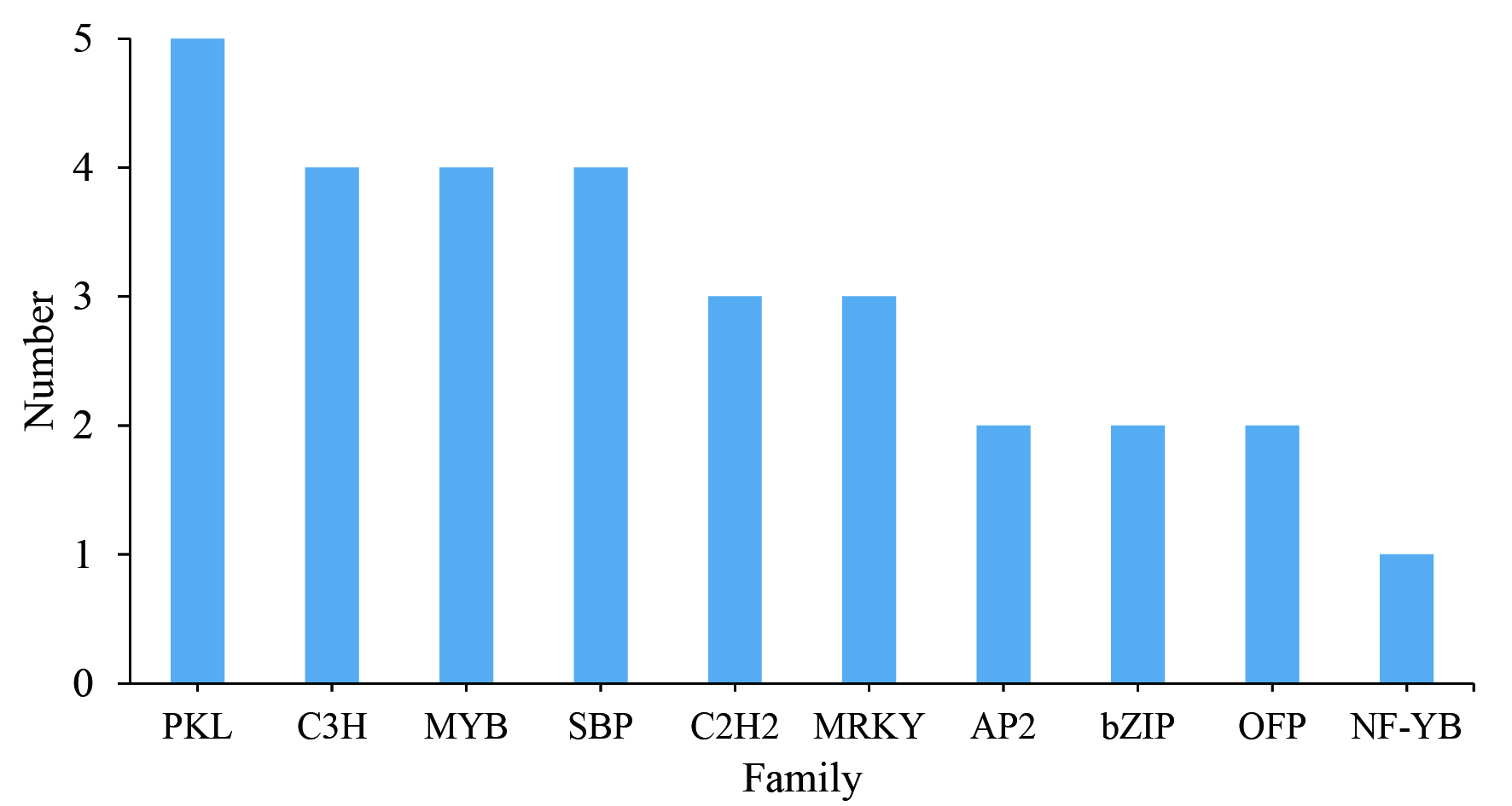

Supplement: Supplementary Figure 2 — The distribution of identified transcription factors during S. tonkinensis kernel development. [file Image_2.JPEG]
